# Determinants of suicidal ideation among patients with mental disorders visiting psychiatry outpatient unit in Mekelle town, psychiatric clinics, Tigray, Northern Ethiopia: a case–control study

**DOI:** 10.1186/s12991-020-00270-x

**Published:** 2020-03-12

**Authors:** Abreha Tsegay, Ashenafi Damte, Adam Kiros

**Affiliations:** Department of Psychiatric Nursing, School of Nursing, College of Health Sciences, Mekelle University, Mekelle, Tigray Ethiopia

**Keywords:** Suicidal ideation, Mental disorder, Determinant, Hospital, Northern Ethiopia

## Abstract

**Background:**

Globally, more than 450 million people suffer from a mental or behavioral disorder. Psychiatric disorder and its duration, physical illness, family history of mental illness, previous suicidal attempt, unemployment, poor social support, and psychotic symptoms are of the main reasons enabling patients to be suicidal ideates. The purpose of this study is to identify the determinants of suicidal ideation among patients with mental disorders in Mekelle, Ethiopia.

**Methods:**

Case–control study design was employed with a total of 221 study subjects (74 cases and 147 controls) in Mekelle, Ethiopia. Suicidal ideation was measured by the Suicidal Behavior Questionnaire-Revised (SBQ-R) scale. Bivariate and multiple logistic regression analyses were performed to determine between the independent and dependent variables.

**Results:**

This study revealed that family suicide history (AOR = 6.87, 95% CI [1.138–41.531, *P* = 0.036), previous attempts history (AOR = 27.457, 95% CI 10.417–72.368, *P* = 0.0001), family mental illness history (AOR = 3.029, 95% CI 1.088–8.431, *P* = 0.034), major depressive disorders (AOR = 11.182, 95% CI 2.382–52.501, *P* = 0.002), and psychiatric comorbid disorders (AOR = 12.245, 95% CI 1.898–78.986, *P* = 0.008) were significant factors of suicidal ideation.

**Conclusion:**

Family mental illness history, family suicide history, previous suicide attempt history, major depressive disorders, and psychiatric comorbid disorders were significant factors of suicidal ideation.

## Introduction

Globally, more than 450 million people suffer from a mental or behavioral disorder; the total number of people living with depression and anxiety disorders in the world is estimated to 322 million (4.4%) and 264 million (3.6%), respectively. These disorders are common mental disorders and suicide is common in mental disorder especially in depressive patients [1].

There are over 35,000 deaths per year in the United States attributed to suicide and is currently ranked the tenth overall cause of death in the United States. Almost 95% of all persons who commit or attempt suicide have a diagnosed mental disorder. Psychiatric disorders like depressive disorders account for 80% of this figure and schizophrenia accounts for 10%. Psychiatric patients’ risk for suicide is 3 to 12 times that of non-patients, but the degree of risk varies, depending on age, sex, diagnosis, and patient status [2]. Suicidal ideation refers to thoughts of serving as the agent of one’s own death and it may vary in seriousness depending on the specificity of suicide plans and the degree of suicidal intent [3].

In America, the study revealed that suicidality was elevated in patients who reported depression or anxiety (AOR = 1.10, *P* = 0.03), and violent behavior (AOR = 2.59, *P* < 0.001) [4]. A similar study was done in USA revealed that anxiety is a predictor of suicide ideation (AOR = 1.49, 95% CI 1.18–1.88, *P* < 0.01). Also disorder like Post-traumatic stress disorder, PTSD (AOR = 4.21); generalized anxiety disorder, GAD (AOR = 1.70, 95% CI 1.18–2.46, *P* < 0.01); specific phobia (AOR = 1.45, 95% CI 1.16–1.81, *P* < 0.01); and social anxiety disorder, SAD (AOR = 1.38, 95% CI 1.10–1.72, *P* < 0.01) were also significant predictors of suicide ideation [5].

A study done in America, Washington DC, revealed that PTSD (AOR = 4.45, 95% CI 2.58–7.67); major depressive disorder, MDD (AOR = 3.63, 95% CI 2.01–6.40) and PTSD with comorbid mental disorder (AOR = 5.71, 95% CI 2.09–23.85) were risk factors for suicidal ideation at *P* < 0.01 [6]. Another study done in Iran, Tehran, reported hypomanic/manic symptoms (95% CI 0.86–0.98, *P* = 0.007) and depression (95% CI 0.87–0.98, *P* = 0.004) were significantly associated with current suicidal ideation [7].

A study conducted in China showed that GAD (AOR = 1.15 95% CI 1.09–1.22), other psychiatric diagnoses including MDD (AOR = 11.9, 95% CI 6.6 ± 21.5, *P* < 0.001) and anxiety disorders (AOR = 8.45 95% CI 15.23–22.75), and bipolar disorders (AOR = 4.42, 95% CI 8.23–12.09) were risk factors that associated with suicidality did not receive MDD diagnosis, whereas risk of suicidality in patients diagnosed with MDD were: female (AOR = 2.28, 95% CI 1.0–5.1, *P* = 0.042) and had anxiety disorder (AOR = 2.24, 95% CI 1.17–4.28, *P* = 0.015) [8].

Study in Lithuania showed that suicidal ideation was independently associated with the use of antidepressants (AOR = 5.4, 95% CI 1.7–16.9) and with current major depressive episode MDD (AOR = 2.90, 95% CI 1.5–5.8) at *P* < 0.05 [9]. In Singapore, the study showed that suicidal ideation was significantly associated with GAD (AOR = 3.7, 95% CI 1.1–12.3, *P* = 0.032) [10] and a study done in Helsinki, Finland, revealed that suicidal ideation was independently predicted by severe depressive disorder (AOR = 4.980, 95% CI 1.856–13.364, *P* = 0.001) and bipolar disorder type II/not otherwise specified (AOR = 3.975, 95% CI 1.260–12.542, *P* = 0.019) [11].

A study conducted in Japan showed that moderate (AOR = 3.98, 95% CI 1.63–9.72, *P* < 0.01) or more severe depression (AOR = 59.62, 95% CI 8.14–436.80, *P* < 0.01) was associated with current suicidal ideation [12], whereas another study in that country, Tokyo reported that panic disorder with agoraphobia (AOR = 2.405, 95% CI 1.269–3.492, *P* = 0.004); obsessive compulsive disorder, OCD (AOR = 2.912, 95% CI 1.511–5.612, *P* = 0.001); PTSD (AOR = 2.048, 95% CI 1.153–3.637, *P* = 0.014) and specific phobia (AOR = 2.038, 95% CI 1.037–4.005, *P* = 0.039), and borderline personality disorder (AOR = 2.523, 95% CI 1.186–5.366, *P* = 0.016) were predictive of suicide behavior (SB) as a whole and appeared to have predictive value for SB, respectively [13].

Another study in Eastern Nepal showed that suicidal ideation was independently related to a diagnosis of depression (AOR = 24.68, 95% CI 2.127–286.273, *P* = 0.013) [14]. According to a study done in Jena, Germany, showed that comorbid depression disorder (AOR = 2.33, 95% CI 1.12–4.84, *P* = 0.023) and depression (AOR = 1.22, 95% CI 1.13–1.32, *P* = 0.0001) predicted concurrent suicidal ideation [15].

In South Africa, the study showed that both death and suicidal ideation were significantly associated with a primary diagnosis of depressive disorder (AOR = 4.72, 95% CI 1.29–17.24, *P* = 0.019 and 6.54, 95% CI 2.45–17.54, *P* < 0.001, respectively). Factors that uniquely predicted death ideation was: primary diagnosis of bipolar disorder (AOR = 4.61, 95% CI 1.43–14.72, *P* = 0.010); and with suicidal ideation being uniquely predicted by a primary diagnosis of borderline personality disorder (AOR = 7.14, 95% CI 1.34–38.46, *P* = 0.021) [16].

A study conducted in Addis Ababa, Ethiopia, revealed that episodes of no positive symptoms (delusion, hallucination) (AOR = 0.378, 95% CI 0.209, 0.683, *P* value < 0.01), and comorbid depression with schizophrenia (AOR = 5.41, 95% CI 2.764–10.60, *P* < 0.001) had association with suicidal ideation [17]. Another study done in Jimma, Ethiopia, showed that MDD (AOR = 4.48, 95% CI 1.95–10.26, *P* = 0.00), and presence of psychiatric diagnosis (like anxiety, other psychiatry disorders) (AOR = 3.84 and 95% CI 1.16–12.67, *P* = 0.03) were the independent predictors of suicidal behaviors [18].

Identifying determinants of suicidal ideation will be helpful to mental health professionals for routine assessing, designing effective prevention and intervention methods that are important in preventing the effect of suicidal ideation and save the patient’s life.

## Methodology

### Study areas and period

This study was conducted in 2019 at Mekelle town, psychiatric clinics (Ayder Comprehensive Specialized Hospital and Mekelle General Hospital). According to the Ethiopian central statistical agency report; the total population in 2011 has been 367,470 (186,045 males and 181,376 females), which is located in Tigray regional state 783 km away from Addis Ababa, the capital city of Ethiopia to the North. Psychiatric services are given by psychiatrists, general practitioner, M.Sc in mental health, B.Sc in psychiatric nursing and clinical psychologists. More than 30 health professionals work in a psychiatry clinic, out of them, two psychiatrists and two clinical psychologists. The current flow of psychiatric patients on average was 946 and 337 patients per month in Ayder Comprehensive Specialized Hospital and Mekelle General Hospital, respectively. Ayder Comprehensive Specialized Hospital had 24 beds for inpatient services, but Mekelle General Hospital has no inpatient service. The study period was from April 15 to May 30, 2019.

### Study design

An institutional-based unmatched case–control study was conducted.

### Source population and study population

#### Source population

All psychiatry outpatient unit visitors were in Mekelle town, psychiatric clinics, Tigray, Northern Ethiopia.

#### Study population

All psychiatric sampled outpatient unit visitors were in Mekelle town, psychiatric clinics, Tigray, Northern Ethiopia.

### Eligibility criteria

#### Inclusion criteria

- Case: psychiatry outpatient unit visitors, clients who had suicidal ideation (scored 8 and above out of 18) and aged 18 years and above, patients attending treatment at psychiatry clinic during the data collection period were included in the study.
- Control: psychiatry outpatient unit visitors, clients who had no suicidal ideation, responded to suicidal behavior questions and scored less than 8 out of 18.

#### Exclusion criteria

Clients who were unable to communicate and unable to sign verbal informed consent and those who had decision incapacity were not be included in the study.

### Sample size and sampling procedure

#### Sample size

The sample size was calculated using a double proportion formula taken from a study conducted in Jimma showed that one of the risk factors for suicidal ideation was having a family history of mental illness (AOR = 2.25) and *P* = 40% [18]. Based on these assumptions, the sample size was calculated as follows: two-sided confidence level = 95%, power = 80%, ratio of cases to controls = 1:2 and odds ratio = 2.25. Then, the desired sample size using Epi info Stata calculation, odds ratio = 1:2, case = 74 and control = 147, the final sample size was 221 (Table 1).

**Table 1:** Sample size calculation of patients with mental disorders visiting psychiatry outpatient unit in Mekelle town, psychiatric clinics, Northern Ethiopia, 2019

	Kelsey	Fleiss	Fleiss w/CC
Cases	*74*	73	80
Controls	*147*	145	160
Total	*221*	218	240

Italic values indicate sample size of cases and controls used for this study

#### Sampling technique and procedure

**Fig. 1:**
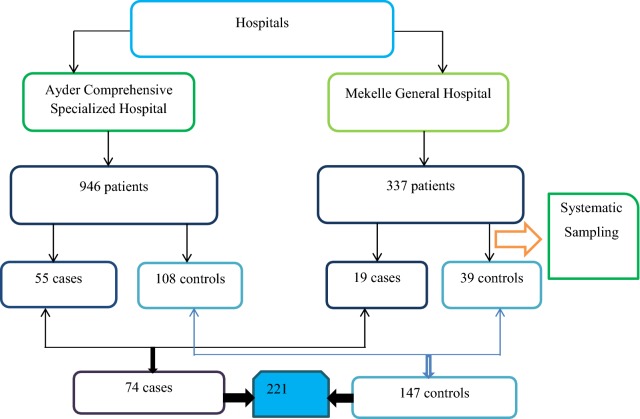
Schematic presentation of the sampling procedure of patients with mental disorders visiting psychiatric outpatient in Mekelle town, psychiatric clinics, Northern Ethiopia, 2019

The systematic sampling technique was employed. The study participants were proportionally allocated in both psychiatric clinics. Since the ratio of case to control was 1:2, every case 2 controls were selected (Fig. 1).

#### Data collection procedure

Face-to-face interview method using a structured questionnaire was used in this study to identify determinants of suicidal ideation such as (1) sociodemographic information, (2) biopsychosocial (3) suicidal ideation, (4) suicidal attempts and its methods. Also, the chart was reviewed to check the psychiatric and other medical illness diagnoses.

The SBQ-R is a self-report measure of suicidal ideation. This shortened version of the SBQ consists of four questions and used a Likert type scale to assess suicidal behavior history, current suicide status and self-appraisal and expectancies about the future likelihood of engaging in suicidal behavior. The magnitude of overall suicidal ideation and behaviors (as defined by SBQ-R a total score ≥ 8 for adult clinical population) and the total score ranges from 3 to 18.

Data were collected by five B.Sc psychiatry professionals having a previous experience of data collection. The principal investigator checked the filled questionnaires for consistency and completeness daily. The questionnaire was translated from English to Tigrigna language by expert and retranslated to English by another proficient in English. This primary version was made to compare with the original English version to resolve inconsistencies and then the data collectors, who are Tigrigna native speakers, collected data in the Tigrigna questionnaire.

### Study variables

#### Dependent variables

Suicidal ideation.

#### Independent variables

Socio-demographic characteristics, medical and substance-related characteristics, mental disorders, illness and hospital related characteristics, psychosocial-related characteristics, suicidal attempt-related characteristics.

### Operational definitions

Suicidal ideation: after the onset of the illness, the patient experiences the degree of having wished, ideation, expectancies about the future likelihood of engaging in suicidal behavior, and plan to kill oneself, and based on SBQ-R scale scores ≥ 8 from 18, at least 2 points out of 4 from suicidal thought or attempt question 1 and the total scale scores range from 3 to 18 [19].

### Data quality assurance

The pre-test was conducted on a sample of 5% (7 controls and 4 cases) of the total study population in Wukiro General Hospital before 2 weeks of data collection and a common understanding was reached between the data collectors to avoid inter-rater variability. The pre-test questionnaires were not included in the analysis as part of the main study. Data collection was collected within 30 working days. Regular supervision by the principal investigator was carried out. Each day during data collection, filled questionnaires’ were checked for completeness and consistency. Incomplete and unclear questionnaires were returned to the data collectors to get it corrected.

### Data analysis procedure

After data collection, filled questionnaires were coded. The data were entered using Epi data version 4.2 statistical software in order to minimize error that occurs during data entry and exported to SPSS; and analyzed using SPSS version 23. Data cleaning was performed to check for frequencies, accuracy, and consistencies and missed values and variables. The finding of this study was presented using text, figure and tables form from the result of frequencies and crosstabs.

A bivariate logistic regression model analysis was done to see the association between the explanatory and outcome variables. Henceforth, multivariable logistic regression analysis was employed by selecting only variables with a *P*-value < 0.25 in the bivariate analysis. The odds ratio with 95% CI was used to measure the strength between dependent and independent variables at *P*-value < 0.05 to determine the level of statistical significance. Variables with the *P*-value less than 0.05 (i.e., 95% CI) in multivariate regression were declared to be potential predictors for suicidal ideation.

## Result

### Sociodemographic characteristics

In this study, a total of 221 participants were invited and fully participated in the study providing a response rate of 100%. Out of them, 147 and 74 were controls and cases, respectively. Thirty-eight 38(25.9%) controls and 34(45.9%) cases were 18–27 years old. Sixty (40.8%) controls and 41(55.4%) cases were single. A hundred twenty-four (84.4%) non-suicidal participants and 57(77.0%) suicidal participants were Orthodox by religion, and A hundred thirty-six (92.5%) controls and 64(86.5%) cases were Tigray by ethnicity. Twenty-seven (18.4%) controls and 23(31.1%) cases educated college and above. Forty-eight (32.7%) controls and 20(27.0%) cases were unemployed. Sixty-one 61(41.5%) controls and 35(47.3%) cases were living with family. Twenty-one (39.6%) non-suicidal respondents and 10(32.3%) suicidal respondents had income 1001–2500 ETB (Table 2).

**Table 2:** Sociodemographic characteristics of patients with mental disorders visiting psychiatry outpatient unit in Mekelle town, psychiatric clinics, Northern Ethiopia, 2019 (*n* = 221, control = 147, and case = 74)

Variable	Category	Control (*n* = 147)	Case (*n* = 74)
Sex	Male	79(53.7)	44(59.5)
	Female	68(46.3)	30(40.5)
Age	18–27	38(25.9)	34(45.9)
	28–37	51(34.7)	22(29.7)
	38–47	36(24.5)	10(13.5)
	≥ 48	22(15.0)	8(10.8)
Marital status	Single	60(40.8)	51(55.4)
	Married	62(42.2)	27(36.5)
	Divorced/widowed	25(17.0)	6(8.1)
Religion	Orthodox	124(84.4)	57(77.0)
	Muslim	20(13.6)	11(14.9)
	Others^**R**^	3(2.0)	6(8.1)
Ethnicity	Tigray	136(92.5)	64(86.5)
	Others^**E**^	11(7.5)	10(13.5)
Educational status	Illiterate	35(23.8)	14(18.9)
	Primary school (1–8)	41(27.9)	16(21.6)
	Secondary school (9–12)	44(29.9)	21(28.4)
	Higher education (12^+^)	27(18.4)	23(31.1)
Occupation	Unemployed	48(32.7)	20(27.0)
	Housewife	22(15.0)	11(14.9)
	Daily laborer	17(11.6)	6(8.1)
	Governmental employee	20(13.6)	13(17.6)
	Farmer	14(9.5)	7(9.5)
	Merchant	8(5.4)	6(8.1)
	Student	10(6.8)	5(6.8)
	Others^**O**^	8(5.4)	6(8.1)
Income	500–1000	12(22.6)	7(22.6)
	1001–2500	21(39.6)	10(32.3)
	2501–3500	4(7.5)	2(6.5)
	3501–5000	8(15.1)	2(6.5)
	> 5000	8(15.1)	10(32.3)
Living condition	Alone	20(13.6)	12(16.2)
	With family	61(41.5)	35(47.3)
	Others^**V**^	66(44.9)	27(36.5)

Others^**E**^ includes Afar and Amhara

Others^**O**^ includes chauffeur, watchman, janitor, pension, and beauty salon

Others^**V**^ includes with a relative, with a spouse, and with prisoners

Others^**R**^ includes Protestant and Catholic

Regarding the educational status, this study found that 112 educated non-suicidal participants and 60 suicidal participants that had an elementary school and above. Of these, 35(31.3%) respondents who had no suicidal ideation and 20(33.3%) respondents who had suicidal ideation had stress-related education. Out of 53 non-suicidal participants and 31 suicidal participants that had a job, 24(45.3%) non-suicidal and 19(61.3%) suicidal had stress in their job. Thirty-nine (26.5%) and 26(35.1%) participants had a history of family/parent/death and were controls and cases, respectively. Among those who had a history of sexual/physical violence, 13(8.8%) and 13(17.6%) had no and had suicidal ideation, respectively. Forty-nine (33.3%) participants who had no suicidal ideation and 34(45.9%) participants who had suicidal ideation had a history of residential change. From those who had a conflict with who had a good relationship, 59(40.1%) and 40(54.1%) were non-suicidal respondents and suicidal respondents, respectively.

### Biopsychosocial characteristics

#### Substance-related characteristics

This study revealed that 15(10.2%) controls and 11(14.9%) cases had a habit of smoking; 15(10.2%) controls and 7(9.5%) cases had a habit of chewing khat; and 21(14.3%) controls and 16(21.6%) cases had a habit of drinking.

Regarding frequency of taking the substance, 3(20.0%) participants that had no suicidal ideation and 7(63.6%) participants that had suicidal ideation often smoked cigarette. Five (33.3%) non-suicidal participants and 5(71.4%) suicidal participants chewed khat. Nine (42.9%) controls and 10(62.5%) cases had drink alcohol sometimes. Talking about duration taking the substance, 7(46.7%) controls and 4(36.4%) cases smoked cigarette for 1–2 years. Only one (6.7%) non-suicidal respondent and 3(42.9%) suicidal respondents chew khat for 2–3 years. Two (9.5%) controls and 8(50.0%) cases had drunk alcohol for 1–2 years (Table 3).

**Table 3:** Substance-related characteristics of patients with mental disorders visiting a psychiatry outpatient unit in Mekelle town, psychiatric clinics, Northern Ethiopia, 2019 (*n* = 85, control = 51, and case = 34)

Variable	Category	Control (*n* = 51)	Case (*n* = 34)
Nicotine	Sometimes	8(53.3)	2(18.2)
	Often	3(20.0)	7(63.6)
	Usually	4(26.7)	2(18.2)
Khat	Sometimes	5(33.3)	5(71.4)
	Often	6(40.0)	0(0.0)
	Usually	4(26.7)	2(28.6)
Alcohol	Sometimes	9(42.9)	10(62.5)
	Often	6(28.6)	2(12.5)
	Usually	6(28.6)	4(25.0)
Nicotine	1–2 years	7(46.7)	4(36.4)
	2–3 years	2(13.3)	0(0.0)
	3–4 years	1(6.7)	2(18.2)
	≥ 4 years	5(33.3)	5(45.5)
Khat	1–2 years	6(40.0)	2(28.6)
	2–3 years	1(6.7)	3(42.9)
	3–4 years	5(33.3)	1(14.3)
	≥ 4 years	3(20.0)	1(14.3)
Alcohol	1–2 years	2(9.5)	8(50.0)
	2–3 years	1(4.8)	3(18.8)
	3–4 years	5(23.8)	2(12.5)
	≥ 4 years	13(61.9)	3(18.8)

#### Medical and psychiatric illness characteristics

**Fig. 2:**
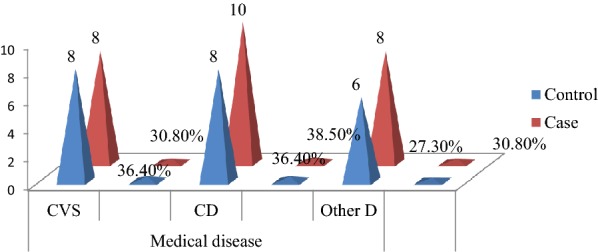
Medical disease comorbid/treated of patients with mental disorders visiting psychiatry outpatient unit in Mekelle town, psychiatric clinics, Northern Ethiopia, 2019 (*n* = 48, control = 22, and case = 26). Physical illness comorbid with psychiatry disorders includes TB (tuberculosis), HIV/AIDS (human immune deficiency virus/acquired immune deficiency syndrome), TB comorbid with HIV/AIDS, and sexually transmitted infection (STI). Cardiovascular system disease (CVS) includes hypertension, diabetes mellitus (DM), and hypertension and DM comorbid, and hypothyroidism. Communicable disease (CD) includes TB, HIV/AIDS; TB and HIV/AIDS comorbid, and STI. Others^**D**^ include breast cancer, burn, internal hemorrhoid, asthma, peptic ulcer disease (PUD), trauma (traumatic brain injury, TBI), hypertension and HIV/AIDS comorbid

Among those psychiatric patients who had comorbid and/or treated physical illness were 22(15.0%) controls and 26(35.1%) cases. Eight (36.4%) respondents who had no suicidal ideation and 8(30.8%) respondents who had suicidal ideation had comorbid cardiovascular diseases. Eight (36.4%) non-suicidal participants and 10(38.5%) suicidal participants had communicable diseases. Three (13.6%) controls and 5(19.2%) cases had hypertension, 4(15.4%) cases had TB, 2(9.1%) controls and 7(26.9%) cases had hypertension comorbid with HIV/AIDS (Fig. 2).

**Fig. 3:**
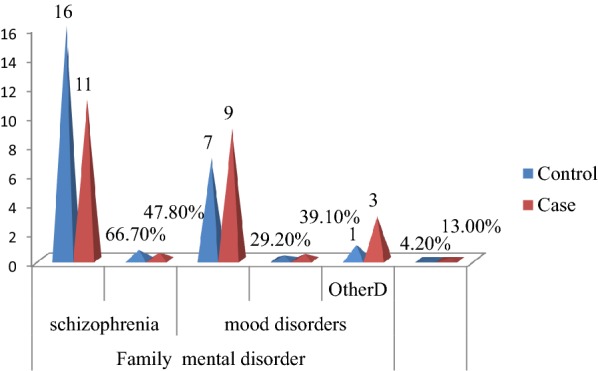
Family mental disorders of patients with mental disorders visiting psychiatry outpatient unit in Mekelle town, psychiatric clinics, Northern Ethiopia, 2019 (*n* = 47, control = 24, and case = 23). Mood disorders include major depressive disorder and bipolar disorder. Others^**D**^ includes intellectual disability, PTSD, OCD, SAD, and GAD

Regarding mental illness, 21(14.3%) participants who had no suicidal ideation and 23(31.1%) participants who had suicidal ideation had a family mental illness history. Two-thirds, 16(66.7%) controls and 11(47.8%) cases had had a family history of schizophrenia. Seven (29.2%) non-suicidal participants and 9(39.1%) suicidal participants had a family history of mood disorder (Fig. 3).

#### Patient’s psychiatric disorders related characteristics

This study showed that diagnosis of patients, 43(29.3%) controls and 38(51.4%) cases were major depressive disorders. Fifty-seven (38.8%) controls and 20(27.0%) cases were schizophrenia. Participants that their diagnosis was psychotic disorder and/or mood disorders with psychotic feature that associated with suicide, 4(3.7%) controls and 11(21.6%) cases had a hallucination that tells/commands him/her to commit suicide. Participants whose diagnosis was either psychotic disorder or major depressive/bipolar I disorder with psychotic feature responded that they had no psychotic features associated with suicide were 102(95.3%) controls 36(70.6%) cases.

Regarding illness onset mode, 63(42.9%) controls and 31(41.9%) cases were their mode of illness onset and duration of illness was less than 3 months. Sixty-eight (46.3%) non-suicidal respondents and 35(47.3%) suicidal respondents, their duration of illness was 3 months to 2 years duration.

Participants’ admission, 34(23.1%) respondents who had no suicidal ideation and 26(35.1%) respondents who had suicidal ideation were admitted and treated as an inpatient at times. Of 26 admitted suicidal participants, 7(26.9%) and 9(34.6%) were admitted and treated as an inpatient at times 1 and 2 weeks, respectively. Seventy (48.3%) non-suicidal respondents and 39 (52.7%) suicidal respondents visited a hospital every 1 month (Table 4).

**Table 4:** Patient’s psychiatric disorders related characteristics of patients with mental disorders visiting psychiatry outpatient unit in Mekelle town, psychiatric clinics, Northern Ethiopia, 2019 (*n* = 221, control = 147, and case = 74)

Variable	Category	Control (*n* = 147)	Case (*n* = 74)
Disorders	Major depressive disorders	43(29.3)	38(51.4)
	Bipolar disorders	21(14.3)	5(6.8)
	Schizophrenia	57(38.8)	20(27.0)
	Other psychotic disorders	9(6.1)	1(1.4)
	Other psychiatric disorders	17(11.6)	10(13.5)
A psychotic feature associated with suicide	No	102(95.3)	36(70.6)
	A hallucination that tells/commands him/her to commit suicide	4(3.7)	11(21.6)
	The delusion that believes I do not want to eat so as to die	1(0.9)	4(7.8)
Mode of illness onset	Abrupt (within hours/days)	13(8.8)	15(20.3)
	Acute (< 3 months)	63(42.9)	31(41.9)
	Insidious, 3–12 months	53(36.1)	19(25.7)
	Insidious, ≥ 12 months	18(12.2)	9(12.2)
Duration of illness since diagnosed	Acute < 3 months	20(13.6)	11(14.9)
	Sub-acute, 3 months–2 years	68(46.3)	35(47.3)
	Chronic, 2 years and above	59(40.1)	28(37.8)
Admission	1 week	9(26.5)	7(26.9)
	2 weeks	8(23.5)	9(34.6)
	3 weeks	9(26.5)	5(19.2)
	1 month and above	8(23.5)	5(19.2)
Frequency visit	Less than 1 month	10(6.8)	15(20.3)
	Every 1 month	71(48.3)	39(52.7)
	Every 2 months	48(32.7)	12(16.2)
	Every 3 months	13(8.8)	2(2.7)
	Other^**F**^	5(3.4)	6(8.1)

Major depressive disorders include major depressive disorder, a major depressive disorder with a psychotic feature, and severe major depressive disorder with the psychotic feature. Bipolar disorders include bipolar I disorder, bipolar I disorder with the psychotic feature

Other psychotic disorders include schizophreniform disorder and brief psychotic disorder and substance-induced psychotic disorder. Other psychiatric disorders include somatic symptom disorder GAD, PTSD, MDD with PTSD, schizophrenia comorbid with post-traumatic symptoms, schizophrenia comorbid with depression, SAD, GAD comorbid with SAD, GAD comorbid with MDD; and OCD

Other^**F**^ includes those who visit less frequency follow-up and send their relatives for follow-up

### Social support characteristics

**Fig. 4:**
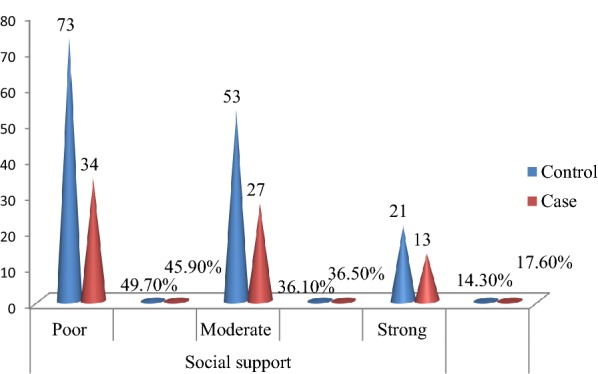
Social support of patients with mental disorders visiting psychiatry outpatient unit in Mekelle town, psychiatric clinics, Northern Ethiopia, 2019 (*n* = 221, control = 147, and case = 74)

This study found that 73(49.7%) controls and 34(45.9%) cases had poor, 53(36.1%) controls and 27(36.5%) cases had moderate social support, respectively (Fig. 4).

### Suicide assessment and related characteristics

#### Suicidal Ideation Assessment (SBQ-R)

This study found that 20(13.6%) non-suicidal participants and two-third 50(67.6%) suicidal participants had suicide attempt history. Six 6(4.1%) controls and 20(27.0%) cases had suicidal attempt in their lifetime. 22(15.0%) controls 34(45.9%) cases had one time suicidal ideation in the past year; and 4(2.7%) controls 39(52.7%) cases had suicide threat. Two-third, 50(67.6%) suicidal respondents responded that they will have likelihood of suicide in the future (Table 5).

**Table 5:** Suicidal ideation assessment of patients with mental disorders visiting psychiatry outpatient unit in Mekelle town, psychiatric clinics, Northern Ethiopia, 2019 (*n* = 221, control = 147, and case = 74)

Variable	Category	Control (*n* = 147)	Case (*n* = 74)
Life time suicidal ideation, intention and attempt	Never	117(79.6)	0(0.0)
	Suicidal thought	21(14.3)	4(5.4)
	Intent	3(2.0)	20(27.0)
	Suicide attempts	6(4.1)	50(67.6)
Frequency suicidal ideation in the past year	Never	124(84.4)	11(14.9)
	Once	22(15.0)	34(45.9)
	Twice	0(0.0)	19(25.7)
	3–4 times	1(0.7)	6(8.1)
	≥ 5 times)	0(0.0)	4(5.4)
Suicidal threats	No	143(97.3)	28(37.8)
	Once	4(2.7)	39(52.7)
	Twice and more	0(0.0)	7(9.5)
Likelihood of suicide in the future	Never	94(63.9)	0(0.0)
	No chance at all	46(31.3)	7(9.5)
	Unlikely^**U**^	7(4.8)	17(23.0)
	Likely^**L**^	0(0.0)	50(67.6)

Unlikely^**U**^ includes rather unlikely and unlikely

Likely^**L**^ includes likely, rather likely and very likely

#### Suicide attempt-related characteristics

This study revealed that 4(2.7%) controls and 11(14.9%) cases had a family history of suicide. Only 2(1.4%) non-suicidal respondents and 24(32.4%) suicidal respondents had current thought about killing oneself); and 20(13.6%) non-suicidal participants and three-fourths, 56(75.7%) suicidal participants had previously suicide attempted history. From 24 suicidal respondents that have current suicidal ideation, 14(58.3%) had the intention to kill themselves via poison, fall from a hill, drown, and availability of lethal methods during that time.

Regarding the intention to attempt suicide, 11(64.7%) respondents who had suicidal ideation that intended to kill themselves via falling from hill, drowning, and burning parts of body and poisoning; and 34(60.7%) suicidal participants were attempted at home. Sixteen (80.0%) controls and 28(50.0%) cases were felt guilty after attempt suicide. Thirteen (65.0%) controls and 29(51.8%) cases aborted suicide attempt by family, whereas 17(85.0%), 16(80.0%), 16(80.0%) controls and 34(60.7%) cases attempted suicide at home (Table 6).

**Table 6:** Suicide attempt-related characteristics of patients with mental disorders visiting psychiatry outpatient unit in Mekelle town, psychiatric clinics, Northern Ethiopia, 2019 (*n* = 98, control = 24, and case = 74)

Variable	Category	Control (*n* = 24)	Case (*n* = 74)
Ideas of intention	I hate to live	0(0.0)	4(16.7)
	Felt hang	1(50.0)	6(25.0)
	Others^**M1**^	1(50.0)	14(58.3)
Methods intended to attempt suicide	Hanging	1(50.0)	6(35.3)
	Others^**M2**^	1(50.0)	11(64.7)
Methods of previous suicide attempted	Hanging	4(20.0)	20(35.7)
	Poisoning	11(55.0)	12(21.4)
	Others^**M3**^	5(25.0)	24(42.9)
Number of suicide attempts	1 time	17(85.0)	25(44.6)
	2 times	1(5.0)	14(25.0)
	≥ 3 times	2(10.0)	17(30.4)
Place of attempt	Home	16(80.0)	34(60.7)
	River and forest	3(15.0)	11(19.6)
	Others^**P**^	1(5.0)	11(19.6)
Feeling after attempt	Felt angry	1(5.0)	13(23.2)
	Felt guilty	16(80.0)	28(50.0)
	Indifferent/felt nothing	3(15.0)	15(26.8)
Aborted suicidal attempt	Family	13(65.0)	29(51.8)
	Friends	1(5.0)	8(14.3)
	God	1(5.0)	7(12.5)
	Others^**A**^	5(25.0)	12(21.4)

Others^**M1**^ include felt poison, fall from a hill, drown, and availability of lethal methods during that time

Others^**M2**^ include falling from a hill, drowning, burning parts of body and poisoning

Others^**M3**^ include by traffic accident intentionally and hanging, hanging and poisoning, burning parts of the body by naphtha and hanging, falling from palace and hill

Others^**P**^ includes at school, at the workplace, at hill and palace, at both home and river, at both home and field

Others^**A**^ includes health professionals, someone who travels, harvest near, myself, neighbors, and rope cut

### Determinants of suicidal ideation

#### Sociodemographic determinants

Marital status (being divorced or widowed) is 0.2 times less likely to have suicidal ideation than married (AOR = .20, 95% CI .046–.879). On the other hand, educational status, age, and alcohol were not significantly factors in this study.

#### Biopsychosocial determinants

This study showed that patients who had family mental illness history are 3.03 times more likely to have suicidal ideation than those had not family mental illness history (AOR = 3.029, 95% CI 1.088–8.431), *P* = 0.034) and having family suicide history is 6.87 times more likely to have suicidal ideation than participants without history of family suicide (AOR = 6.87, 95% CI 1.138–41.531, *P* = 0.036). Patients who had previous attempts history are 27.46 times more likely to have suicidal ideation than those patients did not attempt (AOR = 27.457, 95% CI 10.417–72.368, *P* = 0.0001). On the other hand, physical illness was not a significant factor in this study.

#### Psychiatric disorder determinants

This study revealed that major depressive disorders are 11.18 times more likely risk to have suicidal ideation than those patients their diagnosis is bipolar disorders (AOR = 11.182, 95% CI 2.382–52.501, *P* = 0.002). In addition to major depressive disorder, other psychiatric disorders (those who have comorbid diagnosis that is MDD comorbid with PTSD, PTSD, schizophrenia comorbid with PTSD, SAD comorbid with GAD, OCD, GAD comorbid with MDD, schizophrenia comorbid with depression) were 12.25 times more likely risk to have suicidal ideation than patients whose diagnosis is bipolar disorders (AOR = 12.245, 95% CI 1.898–78.986, *P* = 0.008). On the other hand, psychiatric disorders like schizophrenia, other psychotic disorder were not significant factors in this study (Table 7).

**Table 7:** Multiple logistic regression analysis of determinants of suicidal ideation of patients with mental disorders visiting psychiatry outpatient unit in Mekelle town, psychiatric clinics, Northern Ethiopia, 2019 (*n* = 221, control = 147, and case = 74)

Variable	Category	Case	Control	COR (95% CI)	AOR (95% CI)	*P* value
Educational status	Primary school (1–8)	16(21.6)	41(27.9)	.976[.418–2.276]	.471[.122–1.820]	0.275
	Secondary school (9–12)	21(28.4)	44(29.9)	1.193[.531–2.679]	.538[.127–2.288]	0.402
	University or college (12+)	23(31.1)	27(18.4)	2.130[.926–4.897]	1.754[.445–6.913]	0.422
	Illiterate	14(18.9)	35(23.8)	1		
Marital status	Single	41(55.4)	60(40.8)	1.569[.860–2.864]	.780[.264–2.302]	0.652
	Divorced, widow/ed	6(8.1)	25(17.0)	.551[.203–1.497]	.200[.046–.879]	*0.033*
	Married	27(36.5)	62(42.2)	1		
Age	18–27 year	34(45.9)	38(25.9)	2.461[.969–6.250]	3.134[.676–14.521]	0.144
	28–37 year	22(29.7)	51(34.7)	1.186[.458–3.071]	.886[.191–4.118]	0.877
	38–47 year	10(13.5)	36(24.5)	.547[.123–2.434]	.547[.123–2.434]	0.428
	≥ 48 year	8(10.8)	22(15.0)	1		
Alcohol	Yes	16(21.6)	21(14.3)	1.655[.805–3.404]	1.853[.568–6.045]	0.307
	No	58(78.4)	126(85.7)	1		
Physical illness	Yes	26(35.1)	22(15.0)	3.078[1.594–5.943]	2.467[.870–6.993]	0.089
	No	48(64.9)	125(85.0)	1		
Family mental illness	Yes	23(31.1)	21(14.3)	2.706[1.378–5.315]	3.029[1.088–8.431]	*0.034*
	No	51(68.9)	126(85.7)	1		
Family history of suicide attempt	Yes	11(14.9)	4(2.7)	6.242[1.914–20.357]	6.874[1.138–41.531]	*0.036*
	No	63(85.1)	143(97.3)	1		
Suicide attempt history	Yes	56(75.7)	20(13.6)	19.756[9.711–40.189]	27.457[10.417–72.368]	*0.000*
	No	18(24.3)	127(86.4)	1		
Disorders	MDDs	38(51.4)	43(29.3)	3.712[1.275–10.804]	11.182[2.382–52.501]	*0.002*
	Schizophrenia	20(27.0)	57(38.8)	1.474[.490–4.429]	3.568[.732–17.393]	0.116
	Other psychiatric disorders	10(13.5)	17(11.6)	5.600[1.328–23.620]	12.245[1.898–78.986]	*0.008*
	Other psychotic disorder	1(1.4)	9(6.1)	.467[.048–4.584]	1.742[.083–36.669]	0.721
	Bipolar disorders	5(6.8)	21(14.3)	1		

Italic values indicate variables which show significant determinants with suicidal thought at multivariate analysis

MDDs include major depressive disorder, a major depressive disorder with psychotic features and severe major depressive disorder with a psychotic feature

Other psychiatric disorders include GAD, MDD comorbid with PTSD, PTSD, schizophrenia comorbid with PTSD, social phobia, SAD comorbid with GAD, OCD, GAD comorbid with MDD, schizophrenia comorbid with depression, and somatic symptom disorder

Other psychotic disorders include schizophreniform disorder, brief psychotic disorder, and substance-induced psychotic disorder. Bipolar disorders include bipolar I disorder and bipolar I disorder with psychotic features

## Discussion

This study showed that a family history of suicide attempt was a significant predictor of suicidal ideation. This finding is in line with others studies done in Ethiopia, Addis Ababa and Dessie [10, 17]. Also, in this study, having a family mental illness history is a significant predictor of suicidal ideation. This finding was consistent with studies done in Eastern Nepal and Ethiopia, Jimma [13, 18].

This study also revealed that a previous suicide attempt history was significant for suicidal ideation. This finding was consistent with findings in America; Malaysia; Ethiopia, Addis Ababa and Gonder [4, 16].

This study showed that major depressive disorders were a significant predictor of suicidal ideation. This finding was in line with studies done in America; in America, Washington DC; China; Lithuania; Finland, Helsinki; Japan; Germany, Jena; Eastern Nepal; South Africa; and in Ethiopia, Jimma [4, 6, 8, 9, 11, 12, 14–16, 18].

This study also revealed that other psychiatric disorders (those who have a comorbid diagnosis that is MDD comorbid with PTSD, PTSD, schizophrenia comorbid with PTSD, social phobia comorbid with GAD, OCD, GAD comorbid with MDD, schizophrenia comorbid with MDD) were significant factors for suicidal ideation. This finding was consistent with findings in America, Washington DC; Germany, Jena; Ethiopia, Addis Ababa and Jimma [6, 15, 17, 18].

## Conclusion

Marital status, family mental illness, family suicide history, previous suicide attempt, major depressive disorders and other psychiatric disorders that are those comorbid were significant factors of suicidal ideation.

### Recommendation

- Psychiatric professionals should assess patient suicidal risk assessment routinely and should put the diagnosis with suicidal if the client is suicidal so that every professional focuses on treatment besides the medication.
- Educate the family/caregivers of suicidal patients with previous attempts, current suicidal ideation and intention to have closely followed up.
- It is also recommended researchers to conduct further research studies.

## Limitation of the study

Despite providing valuable baseline data, there are also some limitations encountered:

- Case–control nature of the study design: unmatched case–control does not control confounding factors.
- In this study, only adult psychiatry patients were included, so it is difficult to generalize all psychiatry patients because those who were unable to communicate, children and adolescents psychiatry patients; and decision incapacity consent were not included in the study. So, those excluded participants might be highly suicidal.

## Data Availability

All availability of data and material are attached on the manuscript.

## Abbreviations

AOR: Adjusted odds ratio
CI: Confidence interval
COR: Crude odds ratio
DM: Diabetes mellitus
GAD: Generalized anxiety disorder
HIV/AIDS: Human immune deficiency virus/acquired immune deficiency syndrome
MDD: Major depressive disorder
OCD: Obsessive–compulsive disorder
OSSS: Oslo Social Support Scale
PTSD: Post-traumatic stress disorder
SAD: Social anxiety disorder
SBQ-R: Suicidal Behavior Questionnaire-Revised
SBs: Suicidal behaviors
SPSS: Statistical Package for Social Sciences
STI: Sexually transmitted infection
TB: Tuberculosis
TESI: Treatment-emergent suicidal ideation
TWOSI: Treatment worsening suicidal ideation
UBACC: The University of California, San Diego Brief Assessment of Capacity to Consent
WHO: World Health Organization

## References

[1] World Health Organization (WHO) (2017). Depression and other common mental disorders global health estimates.

[2] Sadock BJ, Sadock VA, Ruiz P (2014). Kaplan & Sadock’s synopsis of psychiatry. Behavioral science/clinical psychiatry.

[3] Jacobs DG, Baldessarini RJ, Conwell Y, Horton L, Fawcett JA, Meltzer H, et al. Assessment and treatment of patients with suicidal behaviors. APA Pract Guidel. 2010;1–83.

[4] Hallgren KA, Ries RK, Atkins DC, Bumgardner K, Roy-Byrne P (2017). Prediction of suicide ideation and attempt among substance-using patients in primary care. J Am Board Fam Med.

[5] Bentley KH, Franklin JC, Ribeiro JD, Kleiman EM, Fox RK (2016). Anxiety and its disorders as risk factors for suicidal thoughts and behaviors: a meta-analytic review. Clin Psychol Rev.

[6] Jakupcak M, Cook J, Imel Z, Fontana A, Rosenheck R, McFall M (2009). Posttraumatic stress disorder as a risk factor for suicidal ideation in Iraq and Afghanistan war veterans. J Trauma Stress.

[7] Khosravani V, Mohammadzadeh A, Bastan FS, Amirinezhad A, Amini M (2019). Early maladaptive schemas and suicidal risk in inpatients with bipolar disorder. Psychiatry Res J.

[8] Li H, Luo X, Ke X, Dai Q, Zheng W, Zhang C, Cassidy RM, Soares JC, Zhang X, Ning Y (2017). Major depressive disorder and suicide risk among adult outpatients at several general hospitals in a Chinese Han population. PLoS ONE.

[9] Bunevicius R, Liaugaudaite V, Peceliuniene J, Raskauskiene N, Bunevicius A, Mickuviene N (2014). Factors affecting the presence of depression, anxiety disorders, and suicidal ideation in patients attending primary health care service in Lithuania. Scand J Prim Health Care.

[10] Beyen A, Getachew Y, Kumara P, Birkie M (2018). Prevalence of suicidal ideation and allied factors among patients with depressive disorder visiting psychiatry outpatient unit of Dessie referral hospital, South Wollo, Ethiopia. Int J Allied Med Sci Clin Res.

[11] Aaltonen K, Näätänen P, Heikkinen M, Koivisto M, Baryshnikov I, Arpov B (2016). Differences and similarities of risk factors for suicidal ideation and attempts among patients with depressive or bipolar disorders. J Affect Disord.

[12] Ando S, Kasai K, Matamura M, Hasegawa Y, Hirakawa H, Asukai N (2013). Psychosocial factors associated with suicidal ideation in clinical patients with depression. J Affect Disord.

[13] Hayashi N, Igarashi M, Imai A, Yoshizawa Y, Utsum K, Ishikawa Y (2012). Post-hospitalization course and predictive signs of suicidal behavior of suicidal patients admitted to a psychiatric hospital: a 2-year prospective follow-up study. BMC Psychiatry.

[14] Pokharel R, Lama S, Adhikari BR, Dharan N (2017). Hopelessness and suicidal ideation among patients with depression and neurotic disorders attending a tertiary care centre at Eastern Nepal. J Nepal Health Res Counc.

[15] Teismann T, Lukaschek K, Hiller TS, Breitbart J, Brettschneider C, Schumacher U (2018). Suicidal ideation in primary care patients suffering from panic disorder with or without agoraphobia. BMC Psychiatry.

[16] Naidoo S, Collings SJ (2017). Suicidal and death ideation in a cohort of psychiatry outpatient units: prevalence and risk factors. Psychol Dev Soc J.

[17] Hussien ZN, Solomon H, Yohannis Z, Ahmed AM (2015). Prevalence and associated factors of suicidal ideation and attempt among people with Schizophrenia at Amanuel Mental Specialized Hospital Addis Ababa, Ethiopia. J Psychiatry.

[18] Salelew E, Dube L, Aber M. Suicidal Behaviours among People with Mental Illness at Jimma University Teaching Hospital Psychiatry Clinic, South West Ethiopia. Qual Prim Care. 2016; 24(6):246–55. http://search.ebscohost.com/login.

[19] Rueda-jaimes GE, Corzo-casasadiego JD, Moreno-quijano C, Camacho PA (2017). The validity of the Suicide Behaviors Questionnaire-Revised in patients with short-term suicide risk. Eur J Psychiatry.

